# Safety and Adequacy of Ultrasound-Guided Percutaneous Renal Biopsy in Children: A Single-Center Experience

**DOI:** 10.7759/cureus.24452

**Published:** 2022-04-24

**Authors:** Kausar Illahi Bux, Khemchand N Moorani, Hina Qureshi, Usha Kumari, Faheemullah Khan, Faryal Farooq, Fakhar Abbas, Muhammad Aman, Abdul Moiz Sahito, Faisal Musharraff, Muhammad Sami Alam

**Affiliations:** 1 Radiology, The Kidney Centre, Karachi, PAK; 2 Paediatric Nephrology, National Institute of Child Health, Karachi, PAK; 3 Pathology (Hematology & Blood Bank), The Kidney Centre, Karachi, PAK; 4 Medicine, Dow University of Health Sciences, Karachi, PAK; 5 Radiology, Aga Khan University Hospital, Karachi, PAK; 6 Radiology, Dow University of Health Sciences, Karachi, PAK; 7 Medicine, University of Health Sciences, Lahore, PAK; 8 Radiology, King Faisal Specialist Hospital and Research Centre, Jeddah, SAU

**Keywords:** hematuria, nephrotic syndrome, children, percutaneous, ultrasound-guided biopsy, renal biopsy

## Abstract

Background

Ultrasound-guided percutaneous renal biopsy (PCRB) is a commonly used technique to obtain renal tissue for histopathological diagnosis in children and adolescents. The objectives of this study include determining the indications for renal biopsy, documenting the safety and efficacy of ultrasound-guided PCRB, and documenting its complications along with histopathological findings in children.

Methodology

The Ethical Review Committee approved this cross-sectional study. Data of all children with either nephrotic or nephritic syndrome from January 2017 to September 2020 (at The Kidney Center Post Graduate Training Institute Karachi) who underwent ultrasound-guided PCRB were collected and analyzed. An ultrasonic examination was performed both before and after the biopsy.

Results

During the research period, 104 individuals underwent PCRB. The average age of the children biopsied was 7.44 ± 4.12 years (range = 1-17 years). The most prevalent reason for biopsy was nephrotic syndrome. Almost 94% of PCRBs were effective. Post-biopsy complications were detected in 16 cases, with peri-nephric hematoma being the most prevalent.

Conclusions

In children, ultrasound-guided PCRB can safely be performed under sedation in experienced hands with an automated biopsy gun needle. The use of real-time ultrasound guidance as well as the automated biopsy gun ensures good outcomes.

## Introduction

Renal biopsy through the percutaneous approach was introduced decades ago. With time, the advances of automated biopsy needles and ultrasound guidance made it safer with great diagnostic yield in the treatment of children with renal disease [1,2]. Ultrasound-guided percutaneous renal biopsy (PCRB) with a biopsy gun has become an accepted procedure for the past many years, allowing the safe passage of the needle into the kidney to obtain tissue for histological diagnosis. It is minimally invasive and gives satisfactory diagnostic output [3,4].

The accurate localization of the lower pole of the kidney by ultrasound is essential for the success and safety of the biopsy procedure. Ultrasound guidance along with automated biopsy needles is intended for optimizing the efficacy and safety of the PCRB procedure. This results in considerable improvement in the general safety and complication rate of the procedure [1,3,5,6].

The primary complications faced after ultrasound-guided renal biopsy are hematuria and hematoma due to post-biopsy bleeding [7-9]. Nonetheless, most of the perirenal hematomas are not clinically significant and resolve spontaneously. The main concerns of the procedure are sufficient tissue for histopathological diagnosis and post-biopsy bleeding which are clinically important [10,11]. This study is undertaken to examine the feasibility and safety of this critical procedure in a pediatric population in a resource-poor setting.

## Materials and methods

This cross-sectional study was conducted among children with a clinical diagnosis of nephrotic and nephritic syndrome from January 2017 to September 2020 at The Kidney Centre Post Graduate Training Institute Karachi (TKC-PGTI). Approval from the Ethical Review Committee was taken (No.69-RAD-102018). Data of all children who underwent ultrasound guided PCRB during the study period were collected and analyzed.

Renal biopsy was done from the lower pole of the left kidney by a trained pediatric nephrologist using an 18-gauge automated spring-loaded biopsy needle under ultrasound guidance of a senior radiologist and under general anesthesia in the operation theater.

**Figure 1:**
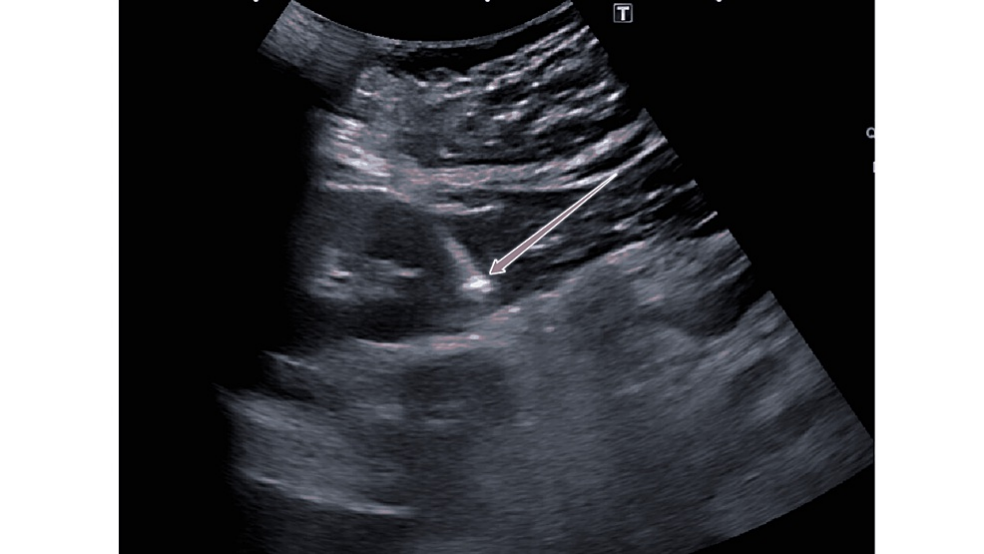
Percutaneous biopsy of the kidney under ultrasound guidance. The sonographic image of the right kidney shows the position of the biopsy needle (arrow) after firing. The entire intraparenchymal portion of the needle is located within the renal cortex, avoiding the renal medulla and more centrally located blood vessels.

Most children had nephrotic syndrome (with steroid resistance or dependence) or frequently relapsing nephrotic syndrome as a primary indication for biopsy. Biopsies were performed using an 18-gauge automated spring-loaded biopsy needle under ultrasound guidance (Figure 1).

All procedures were performed in the operation theater under aseptic measures and general anesthesia along with subcutaneous local anesthesia. All children underwent standard pre-biopsy evaluation including coagulation profile (prothrombin time and partial thromboplastin time), renal function tests (blood urea, creatinine, and electrolytes), and full blood count and ultrasonography for confirmation of both kidneys and exclusion of any congenital anomaly. Blood tests for hepatitis B and C infection were also carried out in all cases. Blood tests for autoimmune markers were done in selected cases.

Post-biopsy, all patients were kept in hospital for at least 24 hours with monitoring of vitals and gross hematuria. Post-biopsy ultrasound was done after six hours for evidence of hematoma or perirenal collection. In case of complication, a follow-up ultrasound was done at 24 hours. The perirenal hematoma or collection was small in all cases and did not warrant intervention. In case of no complication, the ultrasound was repeated a few weeks later. Data were analyzed using Statistical Package for the Social Sciences version 21 (IBM Corp., Armonk, NY, USA), and descriptive statistics were applied.

The abstract of this study was previously presented at the European Congress of Radiology 2019.

## Results

**Figure 2:**
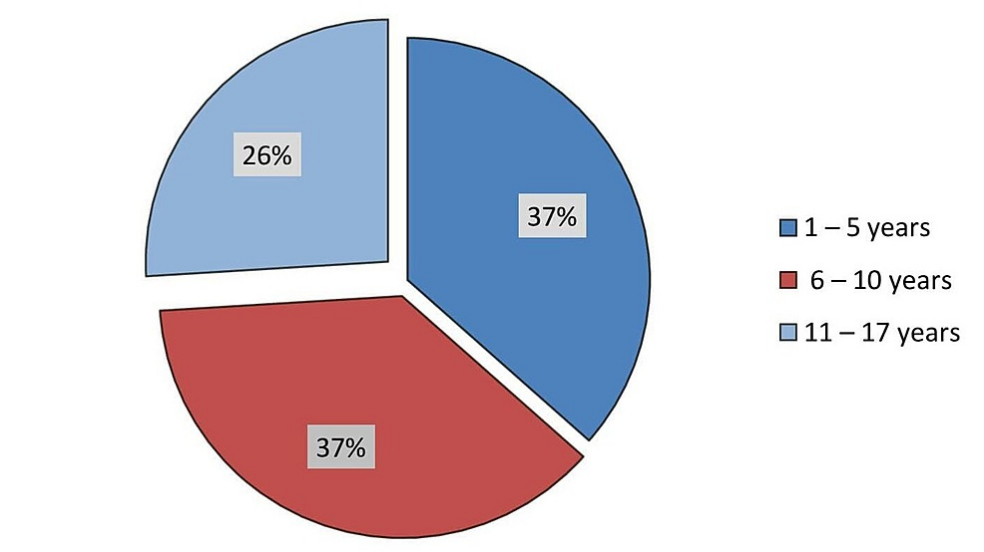
Age distribution of patients. The pie chart shows the age distribution of children undergoing percutaneous renal biopsy.

A total of 104 patients underwent PCRBs during the study period. The mean age of the children biopsied was 7.44 ± 4.12 years (range = 1-17 years) with an almost equal male-to-female ratio (Figure 2).

The most common indication was primary nephrotic syndrome (95.5%). Among nephrotic syndrome, steroid-sensitive nephrotic syndrome was the most common indication for biopsy (n = 54, 51.9%), followed by steroid-dependent nephrotic syndrome (n = 29, 27.8%) and the frequently relapsing type (n = 13, 12.5%). Biopsy for lupus nephritis and rapidly progressing glomerulonephritis/nephritic syndrome was also indicted in two separate cases. Other indications (n = 4, 3.84%) for biopsy included Henoch-Schönlein Purpura and post-infectious glomerulonephritis in two cases each (Table 1).

**Table 1:** Clinical indications of patients who underwent percutaneous renal biopsy.

Clinical diagnosis	Number
Steroid-sensitive nephrotic syndrome	54
Steroid-dependent nephrotic syndrome	29
Frequently relapsing nephrotic syndrome	13
Lupus nephritis	2
Rapidly progressing glomerulonephritis/nephritic syndrome	2
Henoch-Schönlein purpura	2
Post-infectious glomerulonephritis	2
Total	104

Adequate renal tissues were obtained in 98 patients with a success rate of 94%. In the majority of the patients (80%), two core tissue specimens were obtained (Figure 2). Most children (n = 88, 84.62%) underwent a single post-biopsy complication while only two cases showed four (1.92%) post-biopsy complications. An inadequate sample with scanty glomeruli was obtained in six cases.

Almost 94% of the PCRBs were rendered successful. Post-biopsy complications were observed in 16 cases, and perinephric hematoma (12.50%) was the most common post-biopsy complication, followed by gross hematuria (7.77%) (Table 2).

**Table 2:** Complications of percutaneous renal biopsy.

Complications	Number
Perinephric hematoma	13
Renal hematoma	1
Gross hematuria	8
Blood clot/Urine retention	4
Total	26

We observed 13 perinephric hematomas and eight gross hematuria cases, which subsided in three days. However, two post-biopsy gross hematuria complications extended more than three days. None of our patients developed arteriovenous fistula on follow-up Doppler scans. The most commonly diagnosed disease was minimal change disease, followed by focal segmental glomerulosclerosis (Table 3).

**Table 3:** Histopathological diagnosis of patients who underwent percutaneous renal biopsy.

Histopathology	Number
Minimal change disease	42
Focal segmental glomerulosclerosis	33
Membranous glomerular disease	9
Membranoproliferative glomerulonephritis	3
Lupus nephritis	3
Tubulointerstitial nephritis	3
Henoch-Schönlein purpura/Immunoglobulin A nephropathy	3
Post-infectious glomerulonephritis	2
Failed	6
Total	104

## Discussion

Ultrasound-guided PCRB is a recognized and effective diagnostic technique in children and adolescents for many years. Even though there is an abatement in the complication rates and improvement in safety with the use of ultrasound guidance, these procedures have few procedural complications and should not be done without attentiveness and care. The spectrum of complications includes small perirenal and renal hematomas, hemorrhage that may lead to obstruction of the urinary tract, or arteriovenous fistulas. Subcapsular renal hematomas that cause compression over the kidney require primal disclosure by early post-biopsy ultrasound (within 4-24 hours). It has a crucial role in prognosis. The risk of hematuria is increased if the post-biopsy hematoma has a width of 2 cm or more. As reported by the literature and established by different definitions, the complication rate varies between 0% and 45% [1,2,11,12]. A study from Nigeria performed PCRB in 24 patients, of whom complications occurred in two (8.3%) patients. One patient had vague abdominal pain over the biopsy site, while the second had a persistent, painless gross hematuria. The study reported no procedure-related deaths or complications requiring surgical intervention. The authors of the study obtained adequate tissue in 20 patients [1]. Franke et al. reported 99% efficacy of PCRB with no lethal complications. Only three of their patients received a blood transfusion but none of the patients required interventional treatment. The complication rate was 4.1% in their study which is similar to ours [11]. The complication rate should not be above 5% as per the standard of the British Association of Pediatric Nephrology (BAPN). In our study, the complication rate observed is 4.1%, fulfilling the standards of BAPN. No significant difference was seen in the incidence of complications between males and females. Similarly, no statistical difference was seen in the incidence of complications between inpatients and outpatients. Biopsies were followed with ultrasound using a prospective and strict protocol for follow-up examinations, including Doppler ultrasound.

Various sizes and types of needles have been used by different authors to minimize bleeding complications [4,5,12-15]. We used 18-gauge Tru-cut needles with exact positioning of the needles under ultrasound guidance and found that they were safe and effective with no major complications.

In most of our patients, two passes of the needle acquired an adequate sample for histopathology, with only a few requiring three or more passes with a maximum of five passes in one obese patient. We faced a little difficulty with obese patients and those with edema.

Limitations

Our sample size was relatively small and only represented a single center. Our study was a retrospective one, with the inherent limitations of retrospective studies.

## Conclusions

Ultrasound-guided PCRB is a safe approach for acquiring renal tissue for establishing the histopathological diagnosis in children. The use of real-time ultrasound guidance and an automated biopsy gun in experienced hands ensures good outcomes. It provides ample tissue for the diagnosis in more than 90% of the cases.
